# Targeting insulin resistance in type 2 diabetes via immune modulation of cord blood-derived multipotent stem cells (CB-SCs) in stem cell educator therapy: phase I/II clinical trial

**DOI:** 10.1186/1741-7015-11-160

**Published:** 2013-07-09

**Authors:** Yong Zhao, Zhaoshun Jiang, Tingbao Zhao, Mingliang Ye, Chengjin Hu, Huimin Zhou, Zhaohui Yin, Yana Chen, Ye Zhang, Shanfeng Wang, Jie Shen, Hatim Thaker, Summit Jain, Yunxiang Li, Yalin Diao, Yingjian Chen, Xiaoming Sun, Mary Beth Fisk, Heng Li

**Affiliations:** 1 Section of Endocrinology, Diabetes and Metabolism, Department of Medicine, University of Illinois at Chicago, 1819 W. Polk Street, Chicago, IL 60612, USA; 2 Tianhe Stem Cell Biotechnologies Inc., 750 Shunhua Road, Jinan, Shandong 250055, PR China; 3 Section of Endocrinology, General Hospital of Jinan Military Command, 25 Shifan Road, Jinan, Shandong 250031, PR China; 4 Stem Cell Treatment Center, General Hospital of Jinan Military Command, 25 Shifan Road, Jinan, Shandong 250031, PR China; 5 Section of Blood Transfusion, General Hospital of Jinan Military Command, 25 Shifan Road, Jinan, Shandong 250031, PR China; 6 Section of Molecular Diagnostics, General Hospital of Jinan Military Command, 25 Shifan Road, Jinan, Shandong 250031, PR China; 7 Section of Endocrinology, The First Hospital of Hebei Medical University, 89 Donggang Road, Shijiazhuang 050031, PR China; 8 Department of Obstetrics, Jinan Central Hospital, Shandong University, 105 Jiefang Road, Jinan, Shandong 250031, PR China; 9 Texas Cord Blood Bank, 6211 IH-10 west, San Antonio, TX 78201, USA; 10 Department of Neurology, Jinan Central Hospital, Shandong University, 105 Jiefang Road, Jinan, Shandong 250031, PR China

## Abstract

**Background:**

The prevalence of type 2 diabetes (T2D) is increasing worldwide and creating a significant burden on health systems, highlighting the need for the development of innovative therapeutic approaches to overcome immune dysfunction, which is likely a key factor in the development of insulin resistance in T2D. It suggests that immune modulation may be a useful tool in treating the disease.

**Methods:**

In an open-label, phase 1/phase 2 study, patients (N = 36) with long-standing T2D were divided into three groups (Group A, oral medications, n = 18; Group B, oral medications + insulin injections, n = 11; Group C having impaired β-cell function with oral medications + insulin injections, n = 7). All patients received one treatment with the Stem Cell Educator therapy in which a patient’s blood is circulated through a closed-loop system that separates mononuclear cells from the whole blood, briefly co-cultures them with adherent cord blood-derived multipotent stem cells (CB-SCs), and returns the educated autologous cells to the patient’s circulation.

**Results:**

Clinical findings indicate that T2D patients achieve improved metabolic control and reduced inflammation markers after receiving Stem Cell Educator therapy. Median glycated hemoglobin (HbA_1_C) in Group A and B was significantly reduced from 8.61% ± 1.12 at baseline to 7.25% ± 0.58 at 12 weeks (*P =* 2.62E-06), and 7.33% ± 1.02 at one year post-treatment (*P =* 0.0002). Homeostasis model assessment (HOMA) of insulin resistance (HOMA-IR) demonstrated that insulin sensitivity was improved post-treatment. Notably, the islet beta-cell function in Group C subjects was markedly recovered, as demonstrated by the restoration of C-peptide levels. Mechanistic studies revealed that Stem Cell Educator therapy reverses immune dysfunctions through immune modulation on monocytes and balancing Th1/Th2/Th3 cytokine production.

**Conclusions:**

Clinical data from the current phase 1/phase 2 study demonstrate that Stem Cell Educator therapy is a safe approach that produces lasting improvement in metabolic control for individuals with moderate or severe T2D who receive a single treatment. In addition, this approach does not appear to have the safety and ethical concerns associated with conventional stem cell-based approaches.

**Trial registration:**

ClinicalTrials.gov number, NCT01415726

## Background

Type 2 diabetes (T2D) is a major global health issue, with prevalence rates exceeding 12.1% of the population in India, 9.7% in China, and 8.3% in the United States [1,2]. According to a report from the American Diabetes Association (ADA, Philadelphia, PA, USA), the total number of Americans living with diabetes will increase 64% by 2025, and diabetes-related Medicare expenditures will increase by 72% to $514 billion/year. Moreover, diabetes and its associated complications (for example, cardiovascular diseases, stroke, kidney failure and poor circulation) markedly decrease the quality of life, limiting the regular activity and productivity of individuals with the disease and creating significant economic and social burdens [3]. Thus, it is a top priority to find a cure for T2D. To date, animal and clinical studies demonstrate that insulin resistance is the key mechanism leading to the development and pathogenesis of T2D, though many factors are known to contribute to the development and severity of the disease (for example, obesity, genetic factors and sedentary lifestyle) [3]. Several medications have been shown to improve the outcome of T2D treatment through various mechanisms and act on various organs and tissues. However, safety concerns limit the utility of known insulin sensitizers. For example, the peroxisome proliferator-activated receptor-γ (PPAR-γ) agonists (thiazolidinediones, TZDs) are some of the major frontline insulin-sensitizing drugs for clinical treatment of T2D that directly improve insulin sensitivity, but the risk of adverse effects with long-term use of these compounds is a safety concern [4,5]. Alternative approaches are needed.

Increasing evidence reveals that T2D subjects display multiple immune dysfunctions and chronic metabolic inflammation. Specifically, inflammatory cytokines derived from adipocytes and macrophages promote the development of insulin resistance in T2D through JNK and/or IKKβ/NF-κB pathways, including changes in the levels of tumor necrosis factor-α (TNFα), interleukin-1 (IL-1), IL-6, IL-17, monocyte chemoattractant protein-1 (MCP-1), resistin and plasminogen activator inhibitor-1 (PAI-1) [6-10]. Control or reversal of these immune dysfunctions and chronic inflammation may provide an alternative approach for overcoming insulin resistance and may point to a cure for diabetes. However, the failure of several recent clinical trials in Type 1 diabetes (T1D) highlights the challenges we face in conquering the multiple immune dysfunctions by using conventional immune approaches in humans [11-13]. Based on pre-clinical studies in mice and humans [14-17], we have developed Stem Cell Educator therapy [18], an innovative technology designed to control or reverse immune dysfunctions. Stem Cell Educator therapy consists of a closed-loop system that circulates a patient’s blood through a blood cell separator (MCS+, Haemonetics, Braintree, MA, USA), briefly co-cultures the patient’s lymphocytes with adherent cord blood-derived multipotent stem cells (CB-SCs) *in vitro*, and returns the educated lymphocytes (but not the CB-SCs) to the patient’s circulation [18]. Our initial clinical trial in T1D revealed that a single treatment with the Stem Cell Educator provides lasting reversal of immune dysfunctions and allows regeneration of islet β cells and improvement of metabolic control in subjects with long-standing T1D [18,19]. Here, we explore the therapeutic potential of Stem Cell Educator therapy in T2D subjects.

## Methods

### Patients

T2D subjects receiving care through the Section of Endocrinology at the General Hospital of Jinan Military Command (Jinan, Shandong, China) were enrolled in a phase 1/phase 2, open-label clinical trial conducted from August 2011 through September 2012. With oversight from a planning committee, the principal investigator designed the trial and received ethical approval for the clinical treatment protocol and consent from the General Hospital of Jinan Military Command. Written informed consent was obtained from each participant. All subjects receiving Stem Cell Educator therapy had been treated with diet, exercise, oral medications and/or insulin injections at stable doses for at least six months prior to treatment. Key exclusion criteria included clinically significant liver, kidney or heart disease; pregnancy; immunosuppressive medication; viral diseases; or diseases associated with immunodeficiency; or any other clinically significant, coexisting conditions.

### Stem Cell Educator therapy and follow-up

In an open-label, phase 1/phase 2 study, patients (N = 36) with long-standing T2D were divided into three groups (Group A, oral medications, n = 18; Group B, oral medications + insulin injections, n = 11; and Group C having impaired islet β cell function with oral medications + insulin injections, n = 7). Thirty-six participants received a single treatment with the Stem Cell Educator (Tianhe Stem Cell Biotechnology®). The preparation of CB-SC cultures and Stem Cell Educators were performed as previously described [18]. Briefly, a 16-gauge IV needle was placed in the left (or right) median cubital vein, and the patient’s blood was passed through a blood cell separator MCS+ (Haemonetics®, Braintree, MA, USA) for six to seven hours to isolate mononuclear cells in accordance with the manufacturer’s recommended protocol. The collected mononuclear cells were transferred into the device for exposure to allogeneic CB-SCs. CB-SC-treated mononuclear cells were returned to the patient’s circulation via a dorsal vein in the hand with physiological saline. The whole process takes eight to nine hours. Follow-up visits were scheduled 4, 12, 24, 40 and 56 weeks after treatment for clinical assessments and laboratory tests. Previous work demonstrated that participants receiving sham therapy failed to show changes in immune modulation and metabolic control [18]. Thus, the main outcome measures in current trial were changes in glycated hemoglobin (HbA1C) values, islet β-cell function of T2D, and immune markers between baseline and follow-up.

### Efficacy measurements in metabolic control

To determine the insulin sensitivity, we used fasting plasma C-peptide instead of fasting insulin for homeostasis model assessment of insulin resistance (HOMA-IR) and pancreatic islet β-cell function (HOMA-B) analysis, because 1) C-peptide is a by-product of insulin synthesis and released at equal levels and 2) T2D patients received external insulin injections and other treatments that limit the accuracy of HOMA-IR [20,21]. HOMA-IR c-pep was calculated using the equation [20-22]: HOMA-IR c-pep = FPG (mmol/L) × FPC (pmol/L)/22.5. FPG is the value of fasting plasma glucose. FPC is the value of fasting plasma C-peptide. The denominator of 22.5 is a normalizing factor [20]. HOMA-B was calculated using the equation [21,22]: HOMA-B c-pep = 20 × FPC (pmol/L)/(FPG (mmol/L)-3.5).

### Study end points

The primary study end points were feasibility and safety of the Stem Cell Educator therapy through 12 weeks post-treatment and preliminary evaluation of the efficacy of the therapy for change in HbA1C values of T2D through 12 weeks compared to baseline. Pancreatic islet β cell function was assessed by measuring basal and glucose-stimulated C-peptide production over time, as described elsewhere [23,24]. Metabolic control was monitored throughout the study. The secondary study end point was preliminary evidence for efficacy of the therapy in anti-inflammation. Baseline blood samples were collected prior to Stem Cell Educator therapy.

### Flow analysis

Flow analysis was performed as previously described [16]. For cell surface staining, cells were incubated with mouse anti-human monoclonal antibodies (eBioscience, San Diego, CA, USA), including fluorescein isothiocyanate (FITC)-conjugated CD80, phycoerythrin (PE)-conjugated CD86, AF 647-conjugated CD14. For intracellular cytokine staining, cells were initially stained for cell surface antigens (for example, phycoerythrin (PE)-conjugated CD4, FITC-conjugated CD25) and then prepared by using a BD Cytofix/Cytoperm Fixation/Permeabilization kit (BD Biosciences, San Jose, CA, USA). Subsequently, cells were stained with different combinations of antibodies, including FITC-conjugated IL-4, PE-conjugated IL-5, PE-conjugated IL-12, FITC-conjugated IL-13 and FITC-conjugated IL-17A (eBioscience), and Alexa Fluor 647-conjugated anti-Foxp3 (BD Biosciences). Cells were regularly stained for 45 minutes at 4°C and then washed with cold PBS prior to flow analysis. After staining, cells were analyzed using a Cytomics™ FC 500 (Beckman Coulter, Brea, CA, USA) or CyAn ADP (Beckman Coulter, Brea, CA, USA). Isotype-matched rat anti-mouse IgG antibodies (eBioscience) served as a negative control.

### Cytokine assay and ELISA

To prepare for cytokine assay, plasma samples were collected from all subjects before and after (one month) receiving Stem Cell Educator therapy, and kept at −80°C in a refrigerator. To determine cytokine levels, human plasma samples were quantified using commercial ELISA kits following the manufacturer’s instructions. We purchased human IL-1, IL-6, IL-10, TNFα and TGF-β1 ELISA kits from Biolegend, Inc. (San Diego, CA, USA).

### Western blot

CB-SCs were collected and solubilized with Complete Lysis-M buffer with a cocktail of protease inhibitors (Roche Applied Science, Indianapolis, IN, USA). Cell samples (20 μg protein each) were mixed with a loading buffer (62.5 mM Tris–HCl (pH 6.8), 2% SDS, 10% glycerol, 50 mM dithiothreitol (DTT), 2 mg of bromphenol blue) in a volume ratio of 1:1, boiled, loaded and separated by electrophoresis on 10% SDS gel (Bio-Rad, Hercules, CA, USA). The separated proteins were then transferred to a nitrocellulose membrane, blocked with 5% non-fat dry milk in Tris-buffered saline with Tween (TBST) for one hour and incubated with different antibodies: including rabbit anti-human cellular inhibitor of apoptosis protein (cIAP) 1 and cIAP2 monoclonal antibodies (Abcam, Cambridge, MA, USA) and mouse anti-human TNF-RI or TNF-RII monoclonal antibodies (R&D Systems, Minneapolis, MN, USA) at 1:1,000 dilution, diluted in PBST for two hours at room temperature. After washing, the blot was exposed to a horseradish peroxidase-conjugated secondary antibody (1:2,000; Thermo Scientific, Pierce Antibodies, Rockford, IL USA) in PBS-T. The immunocomplexes were visualized by the enhanced chemiluminescence (ECL, GE Healthcare, Waukesha, WI, USA) method. Beta-actin served as an internal loading control.

### TNFα treatment and cell proliferation

To determine the effects of TNFα on the proliferation of CB-SCs, CB-SCs were treated with recombinant human TNFα (R&D Systems) at different doses, such as 100, 50, 25, 12.5 and 0 ng/ml, in non-tissue culture-treated 24-well plates at 37°C, 8% CO_2_ conditions. After three days, cell proliferation was evaluated using a CyQUANTR Cell Proliferation Assay Kit (EMD Millipore Corporation, Billerica, MA, USA) [25]. Cell fluorescence was measured using a Synergy HT Multi-Detection microplate reader (Bio-Tek Instruments Inc., Winooski, VT, USA) equipped with filters for 480 nm excitation and 520 nm emission. The optical values were analyzed using the manufacturer’s software KC4 v3.1.

### Cell sorting and co-cultures

To purify CD14^+^ monocytes, the freshly-isolated peripheral blood mononuclear cells (PBMC) were initially incubated with 2.5% horse serum to block Fc receptor binding and then incubated with FITC-conjugated CD14 (eBiosciences) antibody for 45 minutes at 4°C and subjected to cell sorting using MoFlo (Beckman Coulter, Brea, CA, USA). After confirming the purity of the population (>98%), CD14^+^ monocytes were collected and used in different *in vitro* co-culture experiments with CB-SCs. Culture of CB-SCs were performed as previously described [18]. Purified CD14^+^ monocytes were co-cultured with CB-SCs at a ratio of 1:5 of CB-SCs:monocytes. After co-culture with CB-SCs for 18 hours, floated cells were collected for apoptotic assay (eBiosciences) by flow cytometry.

To determine the molecular mechanisms underlying the interaction between CB-SCs and monocytes, blocking experiments with TNF-RI mAb, TNF-RII mAb and inducible nitric oxide synthase (iNOS) inhibitor 1400W were performed as previously described [15]. Before co-culture with CB-SCs, monocytes were initially stimulated with lipopolysaccharide (LPS, 10 μg/ml) stimulation for 8 hours, and then seeded onto CB-SCs in regular culture medium at a ratio of 1:5 of CB-SCs:monocytes for 48 hrs in the presence or absence of 1400W (100 nM). To block the action of TNF-RI and TNF-RII, the functional grade purified anti-human TNF-RI and TNF-RII monoclonal antibodies (R&D Systems) were administrated at 20 μg/ml in 0.1% BSA/PBS buffer. The 0.1% BSA/PBS buffer-treated wells served as controls. After incubation with CB-SCs at 37°C for two hours, cells were washed with PBS to remove the unused antibodies. The sorted CD14^+^ T cells (1 × 10^5^ cells/ml/well) were seeded onto the TNF-RI or TNF-RII antibody-treated wells in duplicate. To block the action of iNOS and nitric oxide (NO) production, CB-SCs were pre-treated with 1400W (100 nM, Sigma-Aldrich, St. Louis, MO, USA) for 2 hrs, and then co-cultured with LPS-stimulated monocytes for 48 hrs, followed by real time PCR analysis by using Human Th17 for Autoimmunity and Inflammation PCR Array kit (SABiosciences, Valencia, CA, USA).

### Statistical analysis

An intention-to treat approach was used, with 36 patients undergoing Stem Cell Educator therapy. All patients were included in the safety analyses. The primary efficacy end point was the change in HbA1C between baseline and follow-up, with an absolute difference in HbA1C level of at least 0.5% from baseline.

## Results

### Feasibility and safety of Stem Cell Educator therapy in T2D

Baseline characteristics of participants with T2D are provided in Table 1. Thirty-six patients with T2D have received Stem Cell Educator therapy in a safety study, and their results are similar to the safety evaluation with T1D participants [18]. No participants experienced any significant adverse events during the course of treatment and post-treatment for over a year. Patient complaints were limited to mild discomfort during venipunctures at the site of median cubital vein and some soreness of the arm that resolved quickly following aphaeresis.

**Table 1:** Characteristics of the T2D subjects before treatment

**Patient no.**	**Age**	**Gender**	**Marriage**	**History (year)**	**BMI**	**Fasting glucose**	**HbA1C (%)**	C-peptide (ng/ml)*
**Group A: Long-standing patients having normal β cell function with oral medications**								
1	60	M	Yes	13	30.42	10.39	8.6	2.93
2	38	M	Yes	8	20.9	8.39	9.6	0.89
3	53	M	Yes	4	28.72	9.3	7.9	2.07
4	41	F	Yes	4	26.81	8.2	7.8	1.88
5	29	M	Yes	2	26.78	6.2	7.5	1.67
6	43	F	Yes	2	27.55	10.83	8.7	1.93
7	61	M	Yes	8	30.81	6.53	7.4	1.6
8	68	F	Yes	6	21.2	6.21	8.4	0.97
9	40	M	Yes	6	25.31	15.95	12.3	0.8
10	36	M	Yes	1	25.14	11.59	10.2	1.03
11	54	F	Yes	13	28.28	11.95	8.9	0.91
12	55	F	Yes	14	25.04	8.27	9.7	0.91
13	57	M	Yes	1	26.4	6.95	7.5	1.41
14	54	M	Yes	15	32.35	7.6	7.9	1.5
15	45	F	Yes	1	25	9.46	8.4	1.89
16	58	F	Yes	10	22.91	8.97	9.7	1.08
17	57	F	Yes	14	23.88	7.38	7.4	1.07
18	64	M	Yes	13	32.81	8.3	8.4	2.09
**Mean**	**50**			**7.5**	**26.68**	**9.03**	**8.68**	**1.48**
**(SD)**	**(11)**			**(5)**	**(3.46)**	**(2.44)**	**(1.25)**	**(0.58)**
**Group B: Long-standing patients having normal β cell function with insulin injection**								
19	52	M	Yes	12	28.12	10.63	9.4	1.08
20	44	F	Yes	9	24.2	10.64	8.1	0.96
21	60	F	Yes	12	23.23	11.55	10.0	1.13
22	56	M	Yes	11	28.37	12.19	7.5	1.19
23	46	M	Yes	5	29.05	7.62	7.8	1.61
24	44	M	Yes	2	31.35	10.99	8.5	1.96
25	40	M	Yes	4	24.46	9.1	7.4	1.29
26	61	F	Yes	13	30.47	11.03	8.8	1.53
27	64	F	Yes	2	27.06	8.63	8.3	1.64
28	39	M	Yes	11	28.09	10.94	9.8	1.84
29	47	M	Yes	7	26.45	7.27	7.9	1.33
**Mean**	**50**			**8**	**27.35**	**10.05**	**8.5**	**1.41**
**(SD)**	**(9)**			**(4)**	**(2.59)**	**(1.63)**	**(0.9)**	**(0.32)**
**Group C: Long-standing patients having impaired β cell function with insulin injection**								
30	66	F	Yes	18	22.48	10.71	8.3	0.39
31	39	M	Yes	3	19.83	8.0	13.2	0.45
32	55	F	Yes	24	22.27	6.77	7.3	0.16
33	54	M	Yes	15	25.69	8.56	8.5	0.59
34	49	F	Yes	15	24.22	10.15	9.6	0.17
35	54	M	Yes	11	27.72	8.02	8.9	0.56
36	47	M	Yes	15	24.28	9.18	11.1	0.19
**Mean**	**52**			**14**	**23.78**	**8.77**	**9.56**	**0.36**
**(SD)**	**(8)**			**(6)**	**(2.56)**	**(1.36)**	**(1.99)**	**(0.19)**

* The levels of 0.6 to approximately 3.4 ng/ml are the normal ranges for Chinese fasting C-peptide by radioimmunoassay (RIA). To convert C-peptide value to nmol/L, multiply the ng/ml by 0.331.

### Efficacy outcomes in improving metabolic control

**Figure 1:**
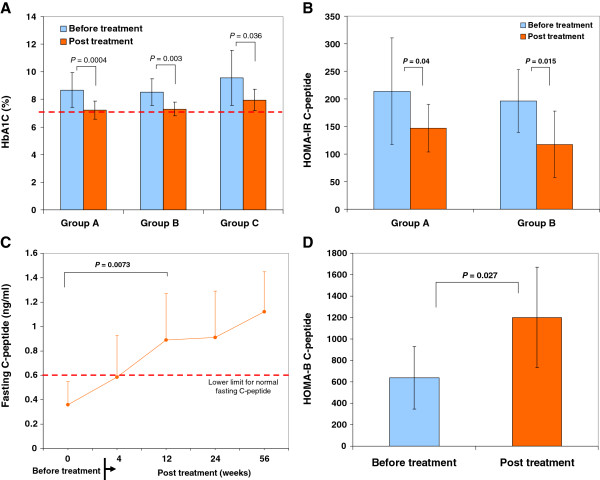
**Improvement of metabolic control by stem cell educator therapy. (A)** Twelve-week follow-up of HbA1C levels in T2D subjects. **(B)** Analysis of insulin sensitivity by HOMA-IR C-peptide at four weeks post-treatment with Stem Cell Educator therapy. **(C)** 56-week follow-up C-peptide levels in Group C T2D subjects with impaired islet β cell function. **(D)** Analysis of islet β cell function by HOMA-B C-peptide at 12-week follow-up post-treatment with Stem Cell Educator therapy in Group C T2D subjects.

After receiving Stem Cell Educator therapy and being discharged from the hospital, patients continued their regular medications. Follow-up studies demonstrated that the median glycated hemoglobin (HbA_1_C) in Group A (n = 18) and Group B (n = 11) was significantly lowered from 8.61% ± 1.12 at baseline to 7.9% ± 1.22 at 4 weeks post-treatment (*P =* 0.026), 7.25% ± 0.58 at 12 weeks post-treatment (*P =* 2.62E-06) (Figure 1A), and 7.33% ± 1.02 at one-year post-treatment (*P =* 0.0002). According to the A1C goal (<7%) recommended by the American Diabetes Association (ADA) for the treatment of adult diabetics, 28% (5/18) of subjects in Group A, 36% (4/11) of subjects in Group B, and 29% (2/7) of subjects in Group C achieved this goal at 12 weeks post-treatment. More than 31% of total subjects achieved and maintained the <7% standard for over a year. Additionally, based on the efficacy criteria, 11 of 18 (61.1%) subjects in Group A, 8 of 11 (72.7%) subjects in Group B, and 4 of 7 (57.1%) subjects in Group C had a reduction of A1C value (>0.5%) at four weeks post-treatment. Thirteen of 18 (72.2%) subjects in Group A, 9 of 11 (81.8%) subjects in Group B, and 6 of 7 (85.7%) subjects in Group C had a reduction of A1C value (>0.5%). Twenty-eight of 36 (78%) of the total subjects decreased A1C levels by 1.28 ± 0.66 at 12 weeks post-treatment. The data demonstrate that glycemic control was improved in T2D patients after Stem Cell Educator therapy.

To explore the change in insulin sensitivity, we analyzed HOMA-IR by the product of fasting plasma glucose and C-peptide (instead of insulin due to subjects receiving insulin injections) in Group A and B. The data revealed that levels of HOMA-IR c-pep were markedly reduced at four weeks follow-up (Figure 1B). It suggests that insulin sensitivity has been improved post-treatment. Consistent with their improved β cell function, the median daily dose of metformin was decreased from 33% to approximately 67%, and insulin was decreased to 35% at 12 weeks post-treatment.

Notably, we found that levels of fasting C-peptide were markedly increased in the long-standing T2D subjects with impaired islet β cell function (Group C, diabetic duration 14 ± 6 years, n = 7, *P =* 0.0073) (Figure 1C). Twelve weeks after receiving the Stem Cell Educator therapy, fasting C-peptide levels reached normal physiological levels and were maintained through the last follow-up for this measure (56 weeks) (0.36 ± 0.19 ng/ml at baseline vs 1.12 ± 0.33 ng/ml at one year post-treatment, *P =* 0.00045, Figure 1C). The β-cell functional analysis by using HOMA-B C-peptide demonstrates that the function of islet β cells was markedly enhanced in group C subjects after receiving Stem Cell Educator therapy (Figure 1D). The data suggest that the restoration of C-peptide may be associated with the regeneration of islet β cells as we demonstrated in our previous work in type 1 diabetes [16,18].

### Efficacy outcomes in correcting the immune dysfunction

**Figure 2:**
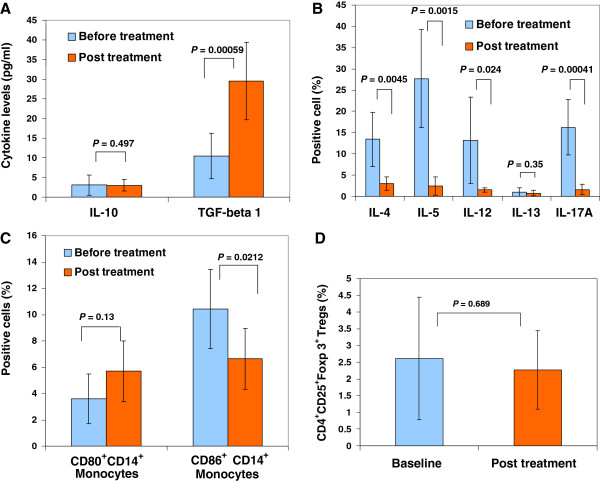
**Anti-inflammatory effects of stem cell educator therapy. (A)** Up-regulation of plasma levels of TGF-β1 in T2D patients at baseline and four weeks after Stem Cell Educator therapy. **(B)** Flow analysis of intra-cellular cytokines demonstrating differential effects on key interleukins at four weeks post-treatment. **(C)** Down-regulation percentage of CD86^+^CD14^+^monocytes in T2D patients at baseline and four weeks after Stem Cell Educator therapy. **(D)** Flow Analysis of CD4^+^CD25^+^Foxp3^+^ Tregs demonstrating no change in the percentage of Tregs at four weeks post-treatment.

To determine the molecular and cellular mechanisms underlying the improvement of metabolic control, we examined the effects of anti-inflammation and immune modulation of Stem Cell Educator therapy in T2D. We used ELISA to examine pro-inflammatory cytokines IL-1, IL-6 and TNFα in the plasma, which are primarily involved in insulin resistance and T2D [8,26]. We found that IL-1, IL-6 and TNFα were all at background levels in these long-standing T2D subjects and failed to show changes after Stem Cell Educator therapy (*P =* 0.557, *P =* 0.316, *P =* 0.603, respectively), probably because metabolic inflammation is a chronic sub-degree inflammation [8] and the plasma samples which were directly collected from the blood of T2D patients, not from the lipopolysaccharide (LPS)-activated monocytes of T2D subjects [27]. Importantly, we found that anti-inflammatory and immune suppressive cytokine TGF-β1 was markedly increased in the plasma of T2D subjects post-treatment at four weeks relative to the baseline levels (Figure 2A). However, IL-10 was unchanged in all participants (*P =* 0.497). These findings suggest up-regulation of TGF-β1 may be one of potential mechanisms contributing to the reversal of insulin resistance by Stem Cell Educator therapy.

Next, using a more sensitive intra-cellular flow cytometry analysis, we examined interleukin-17 (IL-17, also known as IL-17A) and Th1/Th2 immune response-associated cytokines in the peripheral blood of T2D subjects. IL-17A is a well-known pro-inflammatory cytokine involved in autoimmune diseases. Importantly, mounting evidence collected over the past decade indicates that the etiology of T2D includes an autoimmune component that initiates an inflammation affecting pancreatic islet β cells [8,28-32], which provides new insight into the mechanism and potential treatment of insulin resistance through immune modulation. Recent clinical studies showed the increase of circulating Th17 cells and IL-17 production in T2D patients [33] and obese patients [34]. Additionally, recent studies showed that the level of Th1-associated cytokine IL-12 is increased in T2D subjects [35,36]. We found that the production of IL-17, IL-12 and Th2-associated cytokine IL-4 and IL-5 were all markedly decreased after Stem Cell Educator therapy (Figure 2B).

To explore the cellular mechanism underlying the modulation on the Th1/Th2 immune responses, we focused on the changes of co-stimulating molecules CD80/CD86 expressed on the monocytes/macrophages, the professional antigen-presenting cells that play a key role in the onset of chronic inflammation and obesity-associated insulin resistance of T2D [6,37-40]. Flow results demonstrated that the percentage of CD86^+^CD14^+^ monocytes was markedly decreased four weeks after treatment (Figure 2C, *P* = 0.0212). There was no significant change in the level of CD80^+^CD14^+^ monocytes (*P* = 0.13). The ratio of CD86^+^CD14^+^ monocytes/CD80^+^CD14^+^ monocytes was reduced from 3.86 ± 2.56 to 1.22 ± 0.48 (*P =* 0.01). Further flow analysis of the ligands of CD80/CD86, CD28/CTLA-4 expressed on lymphocytes revealed that the expression of CTLA-4 was markedly increased four weeks after receiving Stem Cell Educator therapy (0.51% ± 0.5 before treatment vs 1.98% ± 0.51 post-treatment, *P* = 9.02E-05). However, flow analysis failed to show differences in the expression of co-stimulating molecule CD28 (69.98% ± 14.17 before treatment vs 61.5% ± 10.89 post-treatment, *P* = 0.225). Additionally, we examined changes in the CD4^+^CD25^+^Foxp3^+^ Tregs population after receiving Stem Cell Educator therapy. Flow analysis did not identify any differences between baseline and 4 or 12 weeks post-treatment (Figure 2D, *P* = 0.689). Therefore, these data suggest that Stem Cell Educator therapy may modulate the Th1/Th2 immune responses through the action of antigen-presenting cells monocytes rather than Tregs.

### *In vitro* mechanistic studies of the immune modulation of CB-SCs on monocytes

**Figure 3:**
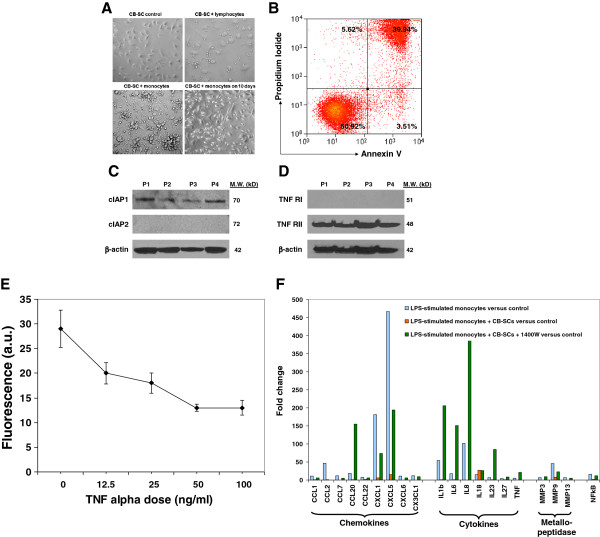
***In vitro *study of the immune modulation of CB-SCs on monocytes. (A)** Phase contrast microscopy shows the co-culture of CB-SC with monocytes (bottom left panel) for 18 hrs. CB-SCs co-culture with lymphocytes (top right panel) served as the control. The impaired CB-SCs after co-culture with monocytes were restored to expansion and became 90 to approximately 100% confluence after 7 to 10 days (bottom right). Original magnification, × 100. **(B)** Apoptotic analysis of floating cells from the co-culture of CB-SCs with monocytes for 18 hrs. **(C)** Western blotting shows the expression of the cellular inhibitor of apoptosis protein (cIAP) 1, not cIAP2, in four preparations of CB-SCs. **(D)** Western blotting shows the expression of tumor necrosis factor receptor II (TNF-RII), not TNF-RI, in four preparations of CB-SCs. **(E)** TNFα suppresses the proliferation of CB-SCs in a dose–response manner. Cell proliferation was evaluated using CyQUANTR Cell Proliferation Assay Kit [25]. **(F)** The blocking experiment with iNOS inhibitor 1400W demonstrates that CB-SC-derived nitric oxide (NO) contributes to the immune modulation of CB-SCs on monocytes. Monocytes were initially stimulated with lipopolysaccharide (LPS, 10 μg/ml) for 8 hrs, and then co-cultured with CB-SCs at ratio 1:5 of CB-SCs:monocytes for 48 hrs in the presence or absence of 1400W (100 nM), followed by real time PCR analysis by using Human Th17 for Autoimmunity and Inflammation PCR Array kit (SABiosciences, Valencia, CA, USA).

To better understand the immune modulation of CB-SC on monocytes, we performed *in vitro* co-culture experiments by using CD14^+^ monocytes purified from human peripheral blood. The purified CD14^+^ monocytes were co-cultured with CB-SCs at different ratios. We found that there were strong reactions after adding the CD14^+^ monocytes to CB-SCs (Figure 3A, bottom left panel). Flow analysis demonstrated that co-culture with CB-SCs for 18 hrs resulted in the significant apoptosis of monocytes at the ratio 1:5 of CB-SC:monocytes (Figure 3B). Correspondingly, both the cell viability and attachment of CB-SCs were also affected in the presence of apoptotic monocytes (Figure 3A, bottom left panel). The cellular processes of CB-SCs were reduced in length, but most were still attached to the bottom (Figure 3A, bottom left panel). Interestingly, these impaired CB-SCs were restored after co-culture for 2 to 3 days; they continually expanded and became 90 to approximately 100% confluence after 7 to 10 days (Figure 3A, bottom right panel). Mechanistic studies revealed that CB-SCs displayed the cellular inhibitor of apoptosis protein (cIAP) 1 [41] that protects CB-SCs against the cytotoxic effects of monocytes, allowing them to survive and proliferate (Figure 3C). To further explore the molecular mechanisms underlying the cytotoxic effects of monocytes on CB-SCs, we found that CB-SCs expressed TNF-RII but not TNF-RI (Figure 3D). Recombinant TNF showed cytotoxicity to CB-SCs at different doses (Figure 3E). Notably, CB-SCs pre-treated with TNF-RII mAb (20 μg/ml) at a ratio of 1:10 could markedly block the toxic action of monocytes and protect 50% of CB-SCs with good cell viability and morphology.

To further explore the immune modulation of CB-SCs on monocytes, LPS-stimulated purified CD14^+^ monocytes were co-cultured with CB-SCs. Real time PCR array showed that co-culture with CB-SC could significantly down-regulate numbers of LPS-stimulated, inflammation-related genes, including chemokines, multiple cytokines and matrix metallopeptidase, along with signaling pathway molecule NF-κB (Figure 3F). These data clearly indicate that *in vitro* co-culture with CB-SCs causes substantial down-regulation of inflammation-associated gene expressions in monocytes. Previous work showed that CB-SCs function as immune modulators on lymphocytes via nitric oxide (NO) production [15]. To confirm the action of NO involved in the immune modulation of CB-SCs on monocytes, the specific inducible nitric oxide synthase (iNOS) inhibitor 1400W was applied to the co-culture system. The data demonstrated that the inhibitory effects of CB-SC on LPS-stimulated monocytes could be significantly reversed in the presence of iNOS inhibitor 1400W (Figure 3F). Interestingly, we found that blocking NO production in CB-SCs could markedly increase the expressions of chemokine CCL20 and cytokines (for example, IL-1β, IL-6, IL-8, IL-23 and TNFα) in monocytes. Thus, it indicates that CB-SC-derived NO plays an essential role in the immune modulating and anti-inflammatory effects of CB-SCs on monocytes.

## Discussion

Insulin resistance is the hallmark of T2D. It is widely accepted that the inability of pancreatic β cells to function in compensating for insulin resistance leads to the onset of clinical diabetes. Persistent metabolic stresses including glucotoxicity, lipotoxicity, chronic metabolic inflammation, oxidative stress and endoplasmic reticulum stress, cause progressive dysfunction of islet β cells and finally lead to the cellular death and absolute shortage of islet β cells in long-standing T2D subjects [42]. The current phase 1/2 study demonstrates the safety and therapeutic efficacy of Stem Cell Educator therapy in the treatment of T2D. Insulin sensitivities were markedly increased after receiving Stem Cell Educator therapy, followed by the significant improvement of metabolic controls in these long-standing T2D patients. Notably, we found that T2D subjects in Group C (with the absolute shortage of islet β cells) significantly improved fasting C-peptide levels and β cell function. These data indicate that Stem Cell Educator therapy may open up a new avenue for the treatment of T2D.

Chronic inflammation of visceral adipose tissue (VAT) is a major contributor to insulin resistance mediated by adipose tissue-released adipokines (for example, IL-6, TNFα, MCP-1 and resistin) [40,43]. Growing evidence strongly demonstrated that an accumulation of macrophages by metabolic stress in the sites of affected tissues (such as vasculature, adipose tissue, muscle and liver) has emerged as a key process in the chronic metabolic-stress-induced inflammation [44]. Monocytes/macrophages, as one type of the professional antigen-presenting cells, play an essential role in controlling the Th1/Th2 immune responses and maintaining homeostasis through the co-stimulating molecules CD80/CD86 and released cytokines. Persistent destructive effects of lipid influx (for example, fatty acids and cholesterol) cause macrophage dysfunctions (including defective efferocytosis and unresolved inflammation), resulting in recruitment and activation of more monocytes/macrophages via MCP-1 and its receptor CCR2 [44]. Consequently, inflammatory cytokines (for example, IL-6 and TNFα) produced by activated macrophages induce insulin resistance in major metabolic tissues [26,44,45]. To prove the action of macrophage in chronic inflammation and insulin resistance in T2D, conditional depletion of CD11c^+^ macrophages or inhibition of macrophage recruitment via MCP-1 knockout in obese mice resulted in a significant reduction in systemic inflammation and an increase in insulin sensitivity [46-48].

To clarify the modulation of Stem Cell Educator therapy on blood monocytes, we found that expression of CD86 and CD86^+^CD14^+^/CD80^+^CD14^+^ monocyte ratios have been markedly changed after receiving Stem Cell Educator therapy in T2D subjects. CD80 and CD86 are two principal co-stimulating molecules expressed on monocytes to skew the immune response toward Th1 or Th2 differentiation through their ligands CD28/CTLA4 [49,50]. Due to the differences of expression levels and binding affinity between CD80 and CD86 with their ligands CD28/CTLA4, it is widely accepted that the interaction of CD86 with CD28 dominates in co-stimulating signals; conversely, the combination of CD80 and CTLA4 governs negative signaling [49-52]. The normalization of the CD86^+^CD14^+^/CD80^+^CD14^+^ monocyte ratio post-treatment may favor the immune balance of Th1/Th2 responses in diabetic subjects. Taken together with our *in vitro* study on the direct interaction between CB-SCs and purified CD14^+^ monocytes, these data indicate that restoration of monocyte functions (such as the expression of CD86, cytokine productions and chemokine productions) mainly contributes to anti-inflammation and reversal of insulin resistance following Stem Cell Educator therapy in T2D subjects.

Increasing animal and clinical evidence demonstrate multiple immune cells contributing to the inflammation-induced insulin resistance in T2D, such as abnormalities of lymphocytes (including T cells, B cells and Tregs [53-57]), neutrophils [58], eosinophils [59], mast cells [60] and dendritic cells (DCs) [61,62]. Specifically, B and T lymphocytes have emerged as unexpected promoters and controllers of insulin resistance [57]. These adaptive immune cells infiltrate into the VAT, releasing cytokines (IL-6 and TNFα) and recruiting more monocytes/macrophages via MCP-1/CCR2 [44]. Finally, this obesity-related inflammation leads to insulin resistance [57,63]. Thus, a major challenge for treatment of T2D is to identify therapeutic approaches that fundamentally correct insulin resistance through targeting the dysfunctions of multiple immune cells. The valuable lessons from intensive research pressure over the past 25 years in T1D [11] highlight the difficulties in overcoming these multiple immune dysfunctions by utilizing conventional immune therapy. Stem Cell Educator therapy functions as “an artificial thymus” that circulates a patient’s blood through a blood cell separator [19], briefly co-cultures the patient’s blood mononuclear cells (such as T cells, B cells, Tregs, monocytes and neutrophils) with CB-SCs *in vitro*. During the *ex vivo* co-culture in the device, these mononuclear cells can be educated by the favorable microenvironment created by CB-SCs through: 1) the action of an autoimmune regulator (AIRE) expressed in CB-SCs [18]; 2) the cell-cell contacting mechanism via the surface molecule programmed death ligand 1 (PD-L1) on CB-SCs [15]; and 3) the soluble factors released by CB-SCs. Previous work [15] and current data indicate that CB-SC-derived NO mainly contributes to the immune modulation on T cells and monocytes. During the passage of monocytes and other immune cells through the device, NO, as a free radical released by CB-SCs, can quickly transmit into their cellular membrane, without the aid of dedicated transporters; 4) correcting the functional defects of regulatory T cells (Tregs) [16]; and 5) directly suppressing the pathogenic T cell clones [17]. During this procedure, both peripheral and infiltrated immune cells in VAT can be isolated by a blood cell separator and treated by CB-SCs, leading to the correction of chronic inflammation, the restoration of the immune balance, and clinical improvements in metabolic control via increasing of insulin sensitivity. Additionally, TGF-β1 is a well-recognized cytokine with a pleiotropic role in immune modulation on multiple immune cells, such as the differentiation and function of Th1/Th2 cells and Tregs, as well as B cells, monocytes/macrophages, dendritic cells, granulocytes and mast cells [64-66]. These immune cells are involved in the inflammation-induced insulin resistance in T2D [53-62]. Therefore, the up-regulation of TGF-β1 level in peripheral blood of T2D subjects is another major mechanism underlying the immune modulation after receiving Stem Cell educator therapy.

During the procedure of Stem Cell Educator therapy, the mononuclear cells circulating in a patient’s blood are collected by a blood cell separator. Additionally, patients are required to move their hips, legs and turn to one side every 15 to 30 minutes during the treatment, in order to mobilize their immune cells from peripheral tissues (including adipose tissues) and organs entering into the blood circulation to be processed by a blood cell separator. Thus, the immune cells both in peripheral blood and in tissues can be isolated by a blood cell separator and treated by CB-SCs. The full blood volume is processed approximately twice during Stem Cell Educator therapy (approximately 10,000 ml whole blood) [18], which ensures a comprehensive approach to modulating essentially all circulating immune cells to address multiple immune dysfunctions and overcome global insulin resistance resulting from a variety of reasons. No other current medications and/or other approaches have yet been shown to achieve this unique therapy success. There are some pathogenic immune cells remaining in tissues and lymph nodes which fail to enter into the blood circulation during the procedure and may escape from the treatment by CB-SCs. These immune cells may migrate into the blood circulation and decrease the therapeutic effectiveness. Therefore, T2D subjects may need additional treatment six to nine months later after receiving the first treatment; however, this is yet to be explored in the phase 3 clinical trial.

We observed that the improvement of islet β cell function (C-peptide levels) progresses slowly over weeks after receiving Stem Cell Educator therapy, not disappearing with the progression of time. We reported similar data in previous T1D trials [18,19]. If Stem Cell Educator therapy only temporarily corrects the immune dysfunctions, the clinical efficacy in metabolic control should disappear soon after receiving Stem Cell Educator therapy, because of the short lifespans of most immune cells, (for example, 5.4 days for neutrophils [67], 3 months for lymphocytes, 1 to 3 days for bone marrow-derived monocytes existing in blood and then migrating into tissues). Previous work demonstrated that CB-SCs showed the marked modulation of Th1-Th2-Th3 cell-related genes, including multiple cytokines and their receptors, chemokines and their receptors, cell surface molecules, along with signaling pathway molecules and transcription factors, as indicated by quantitative real time PCR array [16]. Due to these fundamental immune modulations and induction of immune balance [19], this trial indicates that a single treatment with Stem Cell Educator therapy can give rise to long-lasting reversal of immune dysfunctions and improvement of insulin sensitivity in long-standing T2D subjects.

## Conclusions

The epidemic of diabetes is creating an enormous impact on the global economy, as well as on the health of humans. Overcoming insulin resistance is a major target for the treatment of T2D, and mounting evidence points to the involvement of multiple immune dysfunctions in T2D [3,37,40]. Monocytes/macrophages act as key players contributing to these chronic inflammations and leading to insulin resistance in T2D [6,33,37,39,40]. The current phase 1/phase 2 study demonstrates that Stem Cell Educator therapy can control the immune dysfunctions and restore the immune balance through the modulation of monocytes/macrophages and other immune cells, both in peripheral blood and in tissues, leading to a long-lasting reversal of insulin resistance and a significant improvement in insulin sensitivity and metabolic control in long-standing T2D subjects. These findings are subject to further investigation in large-scale, multi-center clinical trials. This novel approach holds great promise for improving treatment and finding a cure for diabetes, specifically for early-stage diabetics. The advantages of Stem Cell Educator therapy may help diabetics to cope with diabetes-associated complications and improve their quality of life.

## Abbreviations

ADA: American Diabetes Association; AIRE: Autoimmune regulator; BSA: Bovine serum albumin; CB-SCs: Cord blood-derived multipotent stem cells; cIAP 1: Cellular inhibitor of apoptosis protein; CTLA-4: Cytotoxic T-Lymphocyte antigen 4; DCs: Dendritic cells; DTT: Dithiothreitol; ECL: Enhanced chemiluminescence; FITC: Fluorescein isothiocyanate; FPC: Value of fasting plasma C-peptide; FPG: the value of fasting plasma glucose; HbA1C: Glycated hemoglobin; HOMA-B: Homeostasis model assessment of pancreatic islet β-cell function; HOMA-IR: Homeostasis model assessment of insulin resistance; IL-1: Interleukin-1; IL-10: Interleukin-10; IL-17: Interleukin-17; IL-4: Interleukin-4; IL-5: Interleukin-5; IL-6: Interleukin-6; iNOS: Inducible nitric oxide synthase; LPS: Lipopolyssacharide; MCP-1: Monocyte chemoattractant protein 1; NO: Nitric oxide; PAI-1: Plasminogen activator inhibitor-1; PBMC: Peripheral blood mononuclear cells; PBS: Phosphate-buffered saline; PBST: Phosphate-buffered saline/Tween; PD-L1: Programmed death ligand 1; PE: Phycoerythrin; PPARγ: Peroxisome proliferator-activated receptor-γ; RIA: Radioimmunoassay; T1D: Type 1 diabetes mellitus; T2D: Type 2 diabetes mellitus; TBST: Tris-buffered saline with Tween; TGF-β1: Transforming growth factor beta 1; Th: Helper T cells; TNF-RI: Tumor necrosis factor receptor I; TNF- RII: Tumor necrosis factor receptor II; Treg: Regulatory T cells; TZDs: Thiazolidinediones; VAT: Visceral adipose tissue.

## Competing interests

Dr. Zhao, inventor of this technology, led the clinical study and has an investment and a fiduciary role in Tianhe Stem Cell Biotechnology, Inc. (and licensed this technology from University of Illinois). YeZ, SW, JS and YL are employees of Tianhe Stem Cell Biotechnologies, Inc., which might have an interest in the submitted work. All other authors (ZJ, TZ, MY, CH, HZ, ZY, YC, HT, SJ, YD, YiC, XS, MF and HL) have no financial interests that may be relevant to the submitted work.

## Authors’ contributions

YZ and ZJ designed the trial and analyzed the data. YZ drafted the manuscript and obtained the funding. ZY, YeZ, HT and SJ collected data. TZ, MY, CH, HZ, YaC, SW, JS, YL, YD, YC, XS, MF and HL contributed to administrative, technical or material support. All authors had full access to all the data and take responsibility for the integrity of the data and the accuracy of the data analysis. All authors read and approved the final manuscript.

## Pre-publication history

The pre-publication history for this paper can be accessed here:

http://www.biomedcentral.com/1741-7015/11/160/prepub
